# *In Vivo* Nanodetoxication for Acute Uranium Exposure

**DOI:** 10.3390/molecules200611017

**Published:** 2015-06-15

**Authors:** Luis Guzmán, Esteban F. Durán-Lara, Wendy Donoso, Fabiane M. Nachtigall, Leonardo S. Santos

**Affiliations:** 1 Laboratory of Asymmetric Synthesis, Institute of Chemistry and Natural Resources, University of Talca, P.O. Box 747, Talca 3460000, Maule, Chile; E-Mails: lguzman@utalca.cl (L.G.); eduran@utalca.cl (E.F.D.-L.); wdonoso@utalca.cl (W.D.); 2 Nanobiotechnology Division at Talca University, Fraunhofer Chile Research Foundation-Center for Systems Biotechnology, FCR-CSB, P.O. Box 747, Talca 3460000, Maule, Chile; E-Mail: fmanke@utalca.cl; 3 Department of Clinical Biochemistry and Immunohematology, Faculty of Health Sciences, University of Talca, P.O. Box 747, Talca 3460000, Maule, Chile

**Keywords:** chelating agent, uranium, PAMAM dendrimer derivatives, acute intoxication

## Abstract

Accidental exposure to uranium is a matter of concern, as U(VI) is nephrotoxic in both human and animal models, and its toxicity is associated to chemical toxicity instead of radioactivity. We synthesized different PAMAM G4 and G5 derivatives in order to prove their interaction with uranium and their effect on the viability of red blood cells *in vitro*. Furthermore, we prove the effectiveness of the selected dendrimers in an animal model of acute uranium intoxication. The dendrimer PAMAM G4-Lys-Fmoc-Cbz demonstrated the ability to chelate the uranyl ion *in vivo*, improving the biochemical and histopathologic features caused by acute intoxication with uranium.

## 1. Introduction

Uranium (U) is a naturally abundant actinide on Earth, heavily used in many chemical forms in civilian and military industries. Possible accidental exposure to uranium dust or spatters during the mining process to industrial applications and waste disposal is a matter of concern. Whatever its route of entry into the body, uranium reaches the blood and is partly stored in target organs such as bones and kidneys. Uranium is nephrotoxic, in both human and animal models, and its effects have been widely described [1]. Depleted Uranium (DU) has been used in counterweights in airplanes and missiles, as radiation shielding, and in inertial guidance devices. Due to its density and ability to undergo phase transition, depleted uranium is also used for anti-tank armor penetrators and as tank armor. The use of DU munitions in military operations has increased the potential exposure of military and civilian personnel to uranium [2,3]. Several studies indicate that most toxicity associated with DU is due to chemical toxicity and not radioactivity, the health consequences of DU exposure remain unclear [3,4].

Several methods have been explored for uranium removal from nuclear waste by techniques such as adsorption employing modified activated carbon, activated carbon-silica aerogel composite materials, ion-imprinted polymers, polyphenolic compounds, *etc*. [5]. However, there are few important studies describing uranium removal from human body. The described data for uranium detoxication are mainly based on the use of chelating agents as gallic acid, 4,5-dihydroxy-1,3-benzenedisulfonic acid (Tiron), diethylenetriaminepentaacetic acid (DTPA), and 5-aminosalicylic acid (5-AS). However, those have limited use due to low bioavailability, *in situ* stock shortages (*i.e.*, due to limited shelf-life or cost), and toxic side effects [6,7].

Thus, trying to provide an alternative solution for this problem we figure to employ the concept of chelation by dendrimers, which is based on simple coordination chemistry. Evolution of an ideal chelator and chelation therapy could completely remove specific toxic metals from desired sites in the body, which would involve an integrated drug design approach [8]. Dendrimers are highly branched, perfectly monodisperse macromolecules with a precisely controlled chemical structure that were first synthesized by Tomalia *et al*. [9] and Newkome *et al*. [10]. Specific properties of dendrimers have attracted great interest in terms of exploring their potential in biomedical applications including drug carriers [11], vectors for gene transfection [12], and as magnetic resonance imaging (MRI) agents [13]. In addition, they have contributed significantly to the fields of host-guest chemistry [14] and metal complexation [15]. Dendrimer architecture, based on multiplied branches, offers advantages including narrow polydispersity, low viscosity and a high density of surface functionalities compared with equivalent molecular weight linear polymers [16]. Polyamido-amine (PAMAM) dendrimers are commercially available and have been widely investigated from a biomedical point of view. The possibility of attaching functional groups such as primary amines, carboxylates, hydroxyls, *etc.*, to dendritic macromolecules is one of the attractive features of dendrimer nanotechnology, thus, dendrimer terminal groups can be tuned to develop high capacity and selective dendritic ligands that are capable of trapping the uranyl ion for biological applications.

Extensive experience demonstrates that acute and chronic human intoxications with a wide range of metals can be treated with considerable efficiency by the administration of a relevant chelating agent [17]. Thus, a chelating agent forming a stable complex with a toxic metal may shield the metal ion from biological targets, thereby reducing the toxicity, even at times after administration where mobilization has not yet occurred [18]. On this context, we used PAMAM G4 and G5 conjugated with different ligands as arginine-tosyl (Arg-Tos), lysine carboxybenzyl/fluorenylmethyloxycarbonyl (Lys-Cbz Fmoc), lysine- carboxybenzyl (Lys-Cbz), folic acid (FA) and coumarine (Cou) with the aim to study their chelating properties and hemocompatibility. The five ligands were selected according to their chemical structures. These ligands present amine, carbonyl and hydrophobic groups, therefore they could be good candidates to generate a great amount of non-covalent interactions such as hydrogen bonds, hydrophobic bonds and electrostatic interactions with uranyl ions [19].

## 2. Results and Discussion 

The global distribution of uranium contamination has remained a persistent environmental and human health problem for several decades. The specific activity of DU is about 60% that of natural U because of this isotopic difference [20], but it retains the chemical toxicity of natural U as a heavy metal [21,22]. The kidney has long been recognized as the ‘critical’ target of U exposure, *i.e*., the organ first perturbed [23], and is considered the primary target organ following both acute and chronic exposures to soluble U compounds [24,25]. Animal evidence also documents other targets of U exposure, including the bone, reproductive, and central nervous systems [26,27].

### 2.1. Dendrimer Synthesis

Lysine and arginine were coupled to the amine terminals of the PAMAM G4 dendrimers by EDC·HCl and HOBt coupling reaction [28,29]. An excess amount of reagent (1:70) was used in the all three cases to allow maximum possible functionalization. Nevertheless, only 15 Arg-Tos, 44 Lys-Cbz and two Lys-Fmoc-Cbz molecules were found attached to the PAMAM G4 dendrimer, respectively. It is proposed that specific number of molecules attached to PAMAM G4 is determined by the steric hindrance and physicochemical behavior of Arg and Lys.

**Figure 1:**
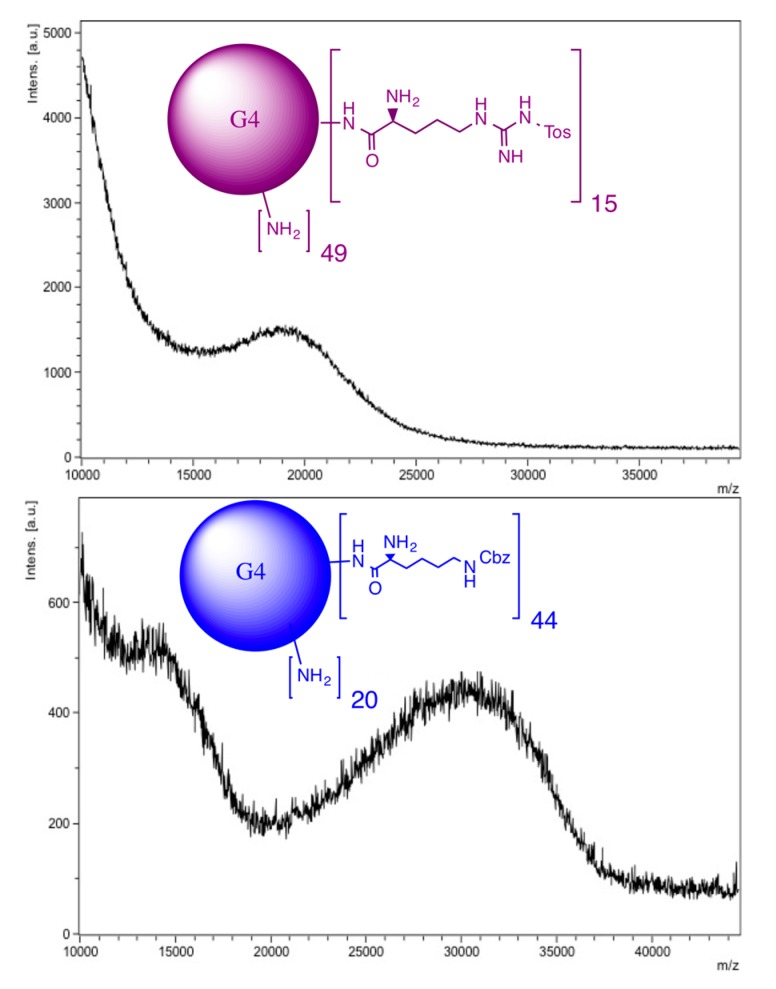
MALDI-MS spectra of PAMAM derivatives G4-(Arg-Tos)_15_ and G4-(Lys-Cbz)_44_.

To avoid dimerization and to increase the degree of conjugation, a Boc synthesis protocol was used. The modifications of the dendrimer surface were characterized by MALDI-TOF spectrometric analysis (Figure 1 and Table 1).

**Table 1:** Full names of ligand and molecular structures of the different PAMAM derivatives synthesized.

Full Name of Ligands	Ligands Molecular Structure
G4-Arg-Tos	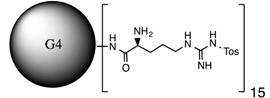
G4-Lys-Fmoc-Cbz	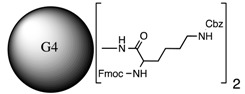
G4-Lys-Cbz	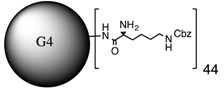
G5-FA	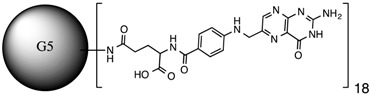
G5-Cou	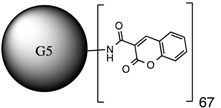

Folic acid and coumarin-3-carboxylic acid were coupled to the amine terminals of the PAMAM G5 dendrimer by EDC·HCl and HOBt coupling reaction [29]. An excess amount of reagents (1:140) was used to allow functionalization of the 128-terminal amine groups of the PAMAM-G5 (MW ~ 28,826) in order to ensure the maximum functionalization. However, only 18 FA (MW of PAMAM G5 + 18 FA ~ 36,800) and 67 Cou molecules (MW of PAMAM G5 + 67 Cou ~ 41,600) were found attached to the G5, respectively. 

It is proposed that the difference in the number of molecules coupled to G5 was due to steric hindrance caused by the size of FA and Cou, respectively. The characteristics of each of them are listed in Table 2.

**Table 2:** Characterization of the synthesized PAMAM G4 and G5 derivatives.

Dendrimer	Molecular Weight	Surface Group	n° Chemical Groups
G4	14,215	Amine	64
G5	28,826	Amine	128
G4-Arg-Tos	19,800	Arginine-Tos	15
G4-Lys-Fmoc-Cbz	15,100	Lysine-Fmoc-Cbz	2
G4-Lys-Cbz	30,500	Lysine-Cbz	44
G5-FA	37,289	Folic Acid	18
G5-Cou	41,566	Coumarine	67

### 2.2. Effects of Derivatized PAMAM on U(VI) Trapping

**Figure 2:**
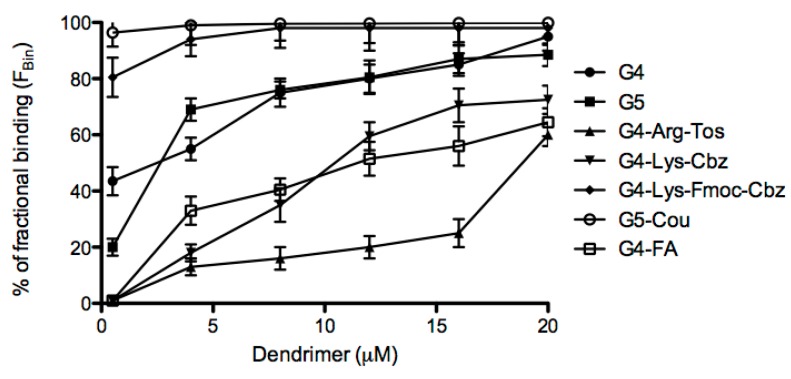
Fractional binding of the different dendrimers synthesized for U(VI).

Each of the dendrimers synthesized was incubated with a solution of U(VI) (20 µM) for 2 h. At the end of the incubation time, the solution was centrifuged and the concentration of the U(VI) unbound to the dendrimer (U_0_) was quantified by spectrofluorimetric methodology. The capture experiment assays were carried out in triplicate for each dendrimer. The F_Bin_ (%) for the commercial G4 and G5 dendrimers showed a capture rate of 95.0% and 88.5%, respectively, at a dendrimer concentration of 20 µM. Decreasing the dendrimer concentration to 0.5 µM, the F_Bin_ affinity reduced to 43.5% and 20.0%, respectively, as depicted in Figure 2. The extent of binding (ExtB) was calculated for that concentration of 0.5 µM showing an ExtB of 17.4 and 8.0, respectively (Table 3). 

**Table 3:** Affinity of synthesized PAMAM dendrimers by U(VI).

	G4	G5	G4-Arg-Tos	G4-Lys-FMoc-Cbz	G4-Lys-Cbz	G5-FA	G5-Cou
C_den_	5.0 × 10^−7^	5.0 × 10^−7^	5.0 × 10^−7^	5.0 × 10^−7^	5.0 × 10^−7^	5.0 × 10^−7^	5.0 × 10^−7^
U_b_	8.7 × 10^−6^	4.0 × 10^−6^	2.0 × 10^−7^	1.6 × 10^−5^	2.0 × 10^−7^	2.0 × 10^−7^	1.9 × 10^−5^
F_Bin_ (%)	43.5	20.0	1.0	80.5	1.0	1.0	96.4
ExtB	17.4	8.0	0.4	32.2	0.4	0.4	38.5

C_den_: concentration of the dendrimer in solution (mol/L), U_b_: uranyl bound to dendrimer (mol/L), F_Bin_ (%): percentage of fraction bound; ExtB: degree of union (mol·U/mol·dendrimer).

The derivatives PAMAM G4-Arg-Tos and G4-Lys-Cbz, at a concentration of 20 µM, showed a lower F_Bin_ than the commercial dendrimers with percentages of 60.0% and 72.5%, respectively, as depicted in Figure 2. However, derivatives PAMAM G4-Lys-Fmoc-Cbz and G5-Cou afforded around 100% of capture of U(IV) at 20 µM (1:1, U(VI):dendrimer, Figure 2). PAMAM G4-Lys-Fmoc-Cbz and G5-Cou showed a high F_Bin_ from 4 to 20 µM (around 100%). To calculate and compare the uranium affinity among all dendrimers in the *in vitro* assays, it was used 0.5 µM dendrimer concentrations, which afforded a 40:1 of U(VI):dendrimer, as depicted in Figure 2 and Table 3. Thus, at 0.5 µM dendrimer concentration, the PAMAM G4-Lys-Fmoc-Cbz and G5-Cou derivatives also presented high percentage of trapping, which achieved around 90% of F_Bin_, and higher extent of binding (Table 3) with 32.2 and 38.5 mol of U(VI) bound per mol/dendrimer, respectively.

Many efforts have been made in order to use dendrimers as chelating agents in aqueous solutions. Diallo *et al*. [30] studied the interaction of different generations of PAMAM dendrimers with U(VI), showing a F_Bin_ of the PAMAM-G5 for uranyl near to 90% at pH 7.0. However, we found a lower F_Bin_ for the PAMAM G5 (20.0%), nevertheless, the different conjugations showed an increased F_Bin_ reaching a 96.4% for the PAMAM G5-Cou and 80.5% in case of the PAMAM G4-Lys-Fmoc-Cbz at a concentration of 0.5 µM, respectively. Furthermore, the conjugation of the PAMAM G5 with coumarin and Lys-Fmoc-Cbz decreased the agglutination of RBCs observed in the PAMAM generations 4 and 5. Recently, Cao *et al*. [31] and Ilaiyaraja *et al*. [32] used different PAMAM generations conjugated with poly(styrene-co-divinylbenzene) adsorbents carrying phosphorus functional groups, or grew a PAMAM G3 on the surface of styrene divinylbenzene, to obtain a chelating resin to adsorb U(VI) from aqueous solution. These studies showed a good correlation in the use of PAMAM dendrimers and the capture of U(VI). However, the insolubility of these resins makes them unsuitable for biological applications, as a good chelating agent for biological applications.

### 2.3. Interaction of the Derivatized Dendrimers with Red Blood Cells (RBCs)

**Figure 3:**
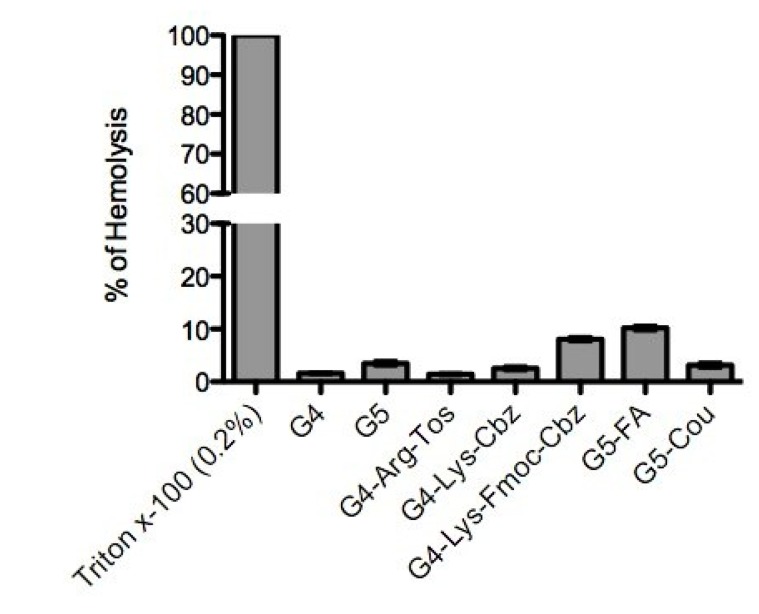
Percentage of hemolysis of red blood cells subjected to a concentration of 4 µM of the different dendrimers studied.

**Figure 4:**
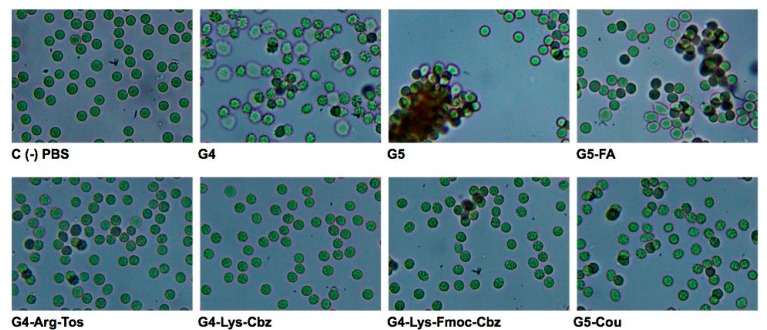
Optical microscopy (400×) of different dendrimers incubated at a concentration of 4 µM, with 2% (*v*/*v*) of washed RBCs.

Figure 3 shows the hemolysis of RBCs expressed as percentage of released hemoglobin compared to the positive control Triton X-100 (0.2%, *v*/*v*). These results indicate that PAMAM dendrimers G5-FA and G4-Lys-Fmoc-Cbz (final concentration of 4 µM in PBS) have the highest percentage of hemolysis (10.2% and 8.1%, respectively). Moreover, Figure 4 shows the differences between negative control (PBS) and the different dendrimers tested. The commercial PAMAM G5 dendrimer produced intense agglutination of RBCs, whereas PAMAM G4 derivatives and the PAMAM G5-Cou showed a reduced agglutination to RBCs when compared with commercial PAMAM G5. Moreover, the commercial PAMAM G4 and the derivative PAMAM G5-FA afforded moderate agglutination of the RBCs as depicted in Figure 4.

Commercial dendrimers PAMAM G4 and G5 showed a good U(VI)-trapping capacity, nevertheless, when incubated with RBCs, these dendrimers caused high agglutination of these cells. Similar results were obtained by Wang *et al.* [33] when the PAMAM G4 dendrimer was examined as a nanocarrier candidate for gene delivery. Low doses of PAMAM G4 dendrimer (10 nM–10 μM~141.3 ng/mL–141.3 μg/mL) caused RBCs aggregation and shape changes of echinocytic, spindle-shaped to spherocyte-like forms, and when the concentration increased to 100 μM (~1.41 mg/mL), PAMAM G4 induced membrane rupture and disintegration [34].

However, dendrimers G4-Arg-Tos and G4-Lys-Cbz showed an entrapment percentage for U(VI) of less than 10%. Furthermore, both dendrimers showed low toxicity with a percentage of hemolysis lower than 10% at a concentration of 4 μM without agglutination of RBCs. Although those dendrimers are not good candidates as chelating agents, this data positions them as good dendrimers for biological assays because they do not affect RBC membranes or cause agglutination thereof.

From the series of dendrimers synthesized G4-Lys-Fmoc-Cbz and G5-Cou had a higher percentage of trapping than commercial dendrimers (G4 and G5) with percentages of trapping of 80.5% and 96.4%, respectively, at a concentration of 0.5 μM, showing that each dendrimer (G4-Lys-Fmoc-Cbz and G5-Cou) is able to capture 32.2 and 45.3 mol of U(VI) per mol of dendrimer in solution, respectively. Moreover, both dendrimer (G4-Lys-Fmoc-Cbz and the G5-Cou) do not cause hemolysis or agglutination of RBCs at a concentration up to 4 μM with no decrease in U(VI) trapping.

### 2.4. In Vivo Effects of the Selected Dendrimers

From all PAMAM dendrimers screened, only PAMAM G4-Lys-Fmoc-Cbz and G5-Cou were selected for *in vivo* studies. As shown in Table 4, after the administration of U(VI) at 5.00 mg/kg, the concentration of urea and uric acid in the plasma of the mice was significantly increased (*p* < 0.001) compared with the control group (saline). However, none of the dendrimers employed, as well as EDTA, caused biochemical changes in these parameters.

**Figure 5:**
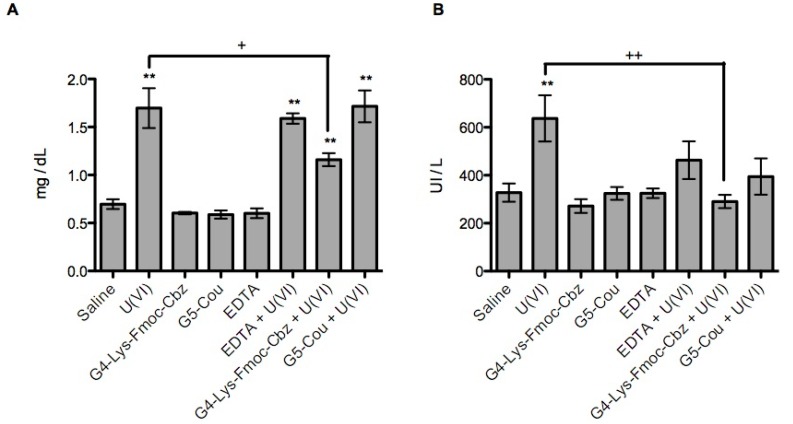
Effectiveness of dendrimers G4-Lys-Fmoc-Cbz and G5-Cou on plasma concentrations of creatinine (**A**) and LDH (**B**). ** *p <* 0.001 compared with the saline control. ^+^ *p <* 0.05; ^++^ *p <* 0.001 compared with the U(VI) group.

The analysis of the plasmatic creatinine showed an elevation in all groups exposed to U(VI) (*p <* 0.001). However, the creatinine concentration in the G4-Lys-Fmoc-Cbz + U(VI) group was significantly lower (*p <* 0.05) compared to the positive control U(VI) (1.2 ± 0.17 *vs.* 1.7 ± 0.5, respectively). This effect was not observed in the group G5-Cou + U(VI) (*p >* 0.05, Figure 5a). According to lactate dehydrogenase (LDH) levels, a wide spectrum indicative of toxicity, it was observed that U(VI) induces a significant increase of enzyme (*p <* 0.05). The same effect was observed in the groups G5-Cou + U(VI) and EDTA + U(VI) (*p <* 0.05). However, the group G4-Lys-Fmoc-Cbz + U(VI) showed no variation according to the saline control (Figure 5b), and was able to maintain the normal levels of LDH with a significant difference between the positive control U(VI) and the group G4-Lys-Fmoc-Cbz + U(VI) (*p <* 0.001). Furthermore, dendrimer derivatives and EDTA did not modify the LDH concentration.

**Table 4:** Plasma concentration of urea, uric acid and calcium in the different study groups.

	Saline	U(VI)	G4-Lys-Fmoc-Cbz	G5-Cou	EDTA	G4-Lys-Fmoc-Cbz + U(VI)	GV5-Cou + U(VI)	EDTA + U(VI)
Urea (mg/dL)	53.3 ± 2.2	226.3 ± 39.4 **	46.3 ± 4.1	43.5 ± 2.2	90.0 ± 17.7	166.3 ± 20.9 **	202.3 ± 31.0 **	192.5 ± 4.4 **
Uric Acid (mg/dL)	0.77 ± 0.15	2.5 ± 0.39 **	1.4 ± 0.21	1.4 ± 0.21	2.2 ± 0.4	3.3 ± 0.16 **	2.8 ± 0.35 **	3.2 ± 0.35 **
Calcium (mg/dL)	7.0 ± 0.23	8.8 ± 0.57 *	7.3 ± 0.42	7.3 ± 0.42	6.8 ± 0.39	8.4 ± 0.31	7.9 ± 0.36	7.7 ± 0.38

* *p <* 0.05, ** *p <* 0.001 compared to saline control.

It is well known that the kidney is the major organ affected by acute U(VI) exposure. According to this study, the administration of 5.00 mg/kg of U(VI) to mice was clearly proved to be nephrotoxic, as indicated by an increase in the concentrations of urea, creatinine and LDH. Moreover, histopathological changes (necrosis) were observed in renal tubular epithelium 48 h after U(VI) injection. These results are in accordance with previous studies describing a series of toxic effects following acute U(VI) exposure [35,36].

Using hematoxylin-eosin staining of kidneys obtained from mice of the different groups, it was observed by optical microscopy that the control (saline) group showed preserved renal tubules and glomeruli architecture. However, the positive control (U(VI)) showed necrotic cells with karyolysis and total eosinophilia of their cytoplasm with dilated tubular atrophy or ‘thyroidization’, which would represent a potential irreversible damage.

**Figure 6:**
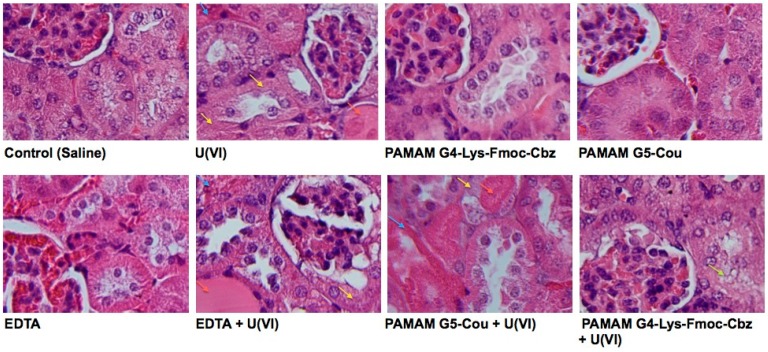
Histological cut of the kidney obtained from the different experimental groups. Yellow arrows: necrosis; blue arrows: eosinophilia; red arrows: thyroidization; green arrows: vesiculation.

The PAMAM G4-Lys-Fmoc-Cbz, G5-Cou, and EDTA groups did not show differences compared to the negative control (saline), highlighting that, together with presenting a low interaction with RBCs and hemolysis, these dendrimers do not damage the kidneys or cause toxicity (as shown by LDH levels) in experimental animals. However, when using these dendrimers to try to neutralize U(VI) in an acute intoxication (5.00 mg/kg) scenario, only the dendrimer PAMAM G4-Lys-Fmoc-Cbz was able to protect the kidney from metal damage (Figure 6). PAMAM G5-Cou + U(VI), and EDTA + U(VI) showed necrosis and karyolysis by eosinophilia and thyroidization like the positive control. However, in the PAMAM G4-Lys-Fmoc-Cbz + U(VI) group a slight increase of eosinophilia in the cytoplasm and vesiculation on the luminal surface was observed, suggesting a protective effect of the derivative PAMAM G4-Lys-Fmoc-Cbz.

It was demonstrated that the administration of 1 μmol/kg of PAMAM G4-Lys-Fmoc-Cbz, promptly after U(VI) exposure, reduced the U(VI)-induced morphological renal changes, which was consistent with low levels of creatinine, urea, LDH and uric acid. In contrast, PAMAM G5-Cou was less effective than PAMAM G4-Lys-Fmoc-Cbz in reducing plasmatic creatinine and urea, as well as attenuating the morphological renal damage induced by U(VI). These results demonstrate that, despite displaying similar behavior *in vitro*, the ability to interact and chelate the U(VI) is not maintained in the same manner *in vivo*, which shows that for biomedical applications the dendrimer PAMAM G4-Lys-Fmoc-Cbz has a better ability to attenuate the clinical and renal damages associated with acute exposure to uranium.

## 3. Experimental Section

### 3.1. Dendrimer Synthesis

#### 3.1.1. PAMAM G4-Arginine-Tos-OH (G4-Arg-Tos)

Conjugation of PAMAM-G4 dendrimer (Dendritech, Midland, MI, USA) with Boc-Arginine (Tos)-OH (Sigma Aldrich Co., St-Louis, MO, USA) was carried out by a condensation between the carboxyl group of arginine and the primary amino group of PAMAM [28]. Thus, Boc-arginine (Tos)-OH (64.0 mg, 0.15 mmol) reacted with 1-[3-(dimethylamino)propyl]-3-ethylcarbodi-imide HCl (EDC·HCl, 150 mg, 2.90 mmol, Sigma Aldrich Co.) and *N*-hydroxybenzotriazole (HOBt, 150 mg, Sigma Aldrich Co.) in a mixture of dry *N*-dimethylformamide (DMF, 2.70 mL, Sigma Aldrich Co.) and dry dimethyl sulfoxide (DMSO, 1.00 mL, Sigma Aldrich Co.), under a nitrogen atmosphere for 1 h. The mixture was added dropwise to a solution of PAMAM G4 (40 mg, 2.81 × 10^−3^ mmol) in water (3.00 mL). The reaction mixture was vigorously stirred for 72 h. The functionalized dendrimer was purified through dialysis membranes with a cut-off of 500 Da (Spectrum Laboratories, Inc., Rancho Dominguez, CA, USA) to remove the excess of the amino acid. Then, the product was lyophilized (Freezone 6, LabConco, Kansas City, MO, USA) and the amount of PAMAM G4-Boc-Arginine (Tos)-OH obtained was 55.0 mg. For Boc deprotection a solution of HCl/dioxane (4.00 mL, 4 M) in a 25.0 mL round-bottom flask equipped with a magnetic stirrer was cooled by an ice-water bath under nitrogen. PAMAM G4-Boc-Arginine (Tos)-OH (0.002 mmol) was added in one portion with stirring. The ice-bath was removed and the mixture was kept stirred for 1 h, until layer chromatography (TLC) indicated that the reaction was complete. The reaction mixture was condensed by rotary evaporation under high vacuum at room temperature. The residue was then washed with dry ethyl ether and collected by filtration. The weight of G4-Arg was 32 mg.

#### 3.1.2. PAMAM G4-Lysine-Fmoc-Cbz (G4-Lys-Fmoc-Cbz)

Conjugation of PAMAM-G4 dendrimer with Fmoc-Cbz-Lysine (Sigma Aldrich Co.) was carried out according to the previously reported method [29,37]. Briefly, Fmoc-Cbz-lysine (123 mg, 0.24 mmol) reacted with EDC·HCl (38.0 mg, 0.24 mmol) and HOBt (33 mg, 0.24 mmol) in a mixture of dry DMF (2.90 mL) and dry DMSO (1.00 mL) under a nitrogen atmosphere for 1 h. The mixture was added dropwise to a solution of PAMAM G4 (50.0 mg, 3.5 × 10^−3^ mmol) in water (5.0 mL). The reaction mixture was vigorously stirred for 72 h. The functionalized dendrimer was purified through dialysis membranes with a cut-off of 500 Da to remove the excess of amino acids. After lyophilization, the weight of G4-Lys-Fmoc-Cbz was 41 mg.

#### 3.1.3. PAMAM G4-Lysine-Cbz-OH (G4-Lys-Cbz)

Conjugation of PAMAM-G4 dendrimer with Boc-Lys-Cbz-OH (Sigma Aldrich Co.) was carried out according to the previously reported method [29,37]. Thus, Boc-Lys-Cbz-OH (56.0 mg, 0.208 mmol) reacted with EDC·HCl (150 mg, 2.90 mmol) and HOBt (150 mg) in a mixture of dry DMF (2.70 mL) and dry DMSO (1.00 mL) under a nitrogen atmosphere for 1 h. The mixture was added dropwise to a solution of PAMAM G4 (30.0 mg, 2.1 × 10^−3^ mmol) in water (3.00 mL). The reaction mixture was vigorously stirred for 72 h. The functionalized dendrimer was purified through dialysis membranes with a cut-off of 500 Da to remove the excess of the amino acid. After the lyophilization, the amount of the PAMAM G4-Boc-Lysine-Cbz-OH obtained was 42.0 mg. Finally, a solution of HCl/dioxane (4.00 mL, 4.0 M) in a 25.0 mL round-bottom flask equipped with a magnetic stirrer was cooled by an ice-water bath under nitrogen, and PAMAM G4-Boc-Lysine-Cbz-OH (0.20 mmol) was added in one portion with stirring. The ice-bath was removed and the mixture was kept stirred for 1 h. TLC indicated that the reaction was completed, the reaction mixture was condensed by rotary evaporation under high vacuum at room temperature. The residue was then washed with dry ethyl ether and collected by filtration. The weight of G4-Lys was 35.0 mg.

#### 3.1.4. PAMAM-G5-Folic Acid (G5-FA)

Conjugation of PAMAM-G5 dendrimer (Dendritech) with folic acid (FA, Sigma Aldrich Co.) was carried out by a condensation between the γ-carboxyl group of FA and the primary amino group of PAMAM G5. Thus, FA (104 mg, 0.23 mmol) reacted with EDC·HCl (150 mg, 1.28 mmol) and HOBt (150 mg), in a mixture of dry DMF (2.70 mL) and dry DMSO (1.00 mL), under a nitrogen atmosphere for 1 h. This mixture was added dropwise to a solution of PAMAM G5 (40.0 mg, 1.38 × 10^−3^ mmol) in water (3.00 mL). The reaction mixture was vigorously stirred for 72 h. The functionalized dendrimer was purified through dialysis (using water) membranes with a cut-off of 500 Da to remove the excess of non-reactive folate. After lyophilization, the weight of G5-FA was 43.0 mg.

#### 3.1.5. PAMAM-G5-Coumarin (G5-Cou)

Conjugation of PAMAM-G5 dendrimer with coumarin-3-carboxylic acid (Cou, Sigma Aldrich Co.) was carried out by a condensation between the carboxyl group of Cou and the primary amino group of PAMAM G5. Thus, Cou (39.5 mg, 0.208 mmol) reacted with EDC·HCl (32.0 mg, 0.208 mmol) in a mixture of dry DMF (2.70 mL) and dry DMSO (1.00 mL) under a nitrogen atmosphere for 1 h. The mixture was added dropwise to a solution of PAMAM G5 (40 mg, 1.38 × 10^−3^ mmol) in water (3.00 mL). The reaction mixture was vigorously stirred for 72 h. The functionalized dendrimer was purified through dialysis (using water) membranes with a cut-off of 500 Da to remove the excess of Cou. After lyophilization, the weight of G5-Cou was 55.0 mg.

### 3.2. MALDI Analysis

To confirm the molecular weight of surface modified dendrimers, mass spectrometric analysis of the dendrimers was performed on a matrix assisted laser desorption/ionization-time of flight (MALDI-TOF) spectrometer (Bruker, Bremen, Germany) with a pulsed nitrogen laser (337 nm), operating in positive ion reflector mode, using 19 kV acceleration voltage and 2,5-dihydroxybenzoic acid (DHB, Sigma Aldrich Co.) as matrix.

### 3.3. Affinity of Derivatized Dendrimers to Uranium

#### 3.3.1. Uranium

Uranyl acetate dihydrate [UO_2_(CH_3_COO)_2_·2H_2_O] was obtained from Merck (Darmstadt, Germany). Uranyl (U(VI)) stock solution (2 × 10^−5^ M, 5.4 ppm) was prepared by dissolving UO_2_(CH_3_COO)_2_·2H_2_O in deionized water. For animal experiments, aliquots of stock solution were diluted with saline to obtain the work solutions. The dose of U(VI) administration to mice is either suitable for the requirement of molar ratio of chelating agent to U(VI) or for U(VI)-induced nephrotoxicity. EDTA was used as the positive control of common therapy for heavy metal chelating agent.

#### 3.3.2. Affinity Assay

For each dendrimer, batch experiments were carried out to determine the extent of binding (ExtB) and fraction bound as a percentage (F_Bin_) of U(VI) in aqueous solutions. The U(VI) concentration was kept constant at 5.4 ppm (2 × 10^−5^ M) in all experiments. Aliquots of U(VI), dendrimer stock solution or phosphate buffer (pH 7.4) were added to each tube to prepare 3.0 mL solutions with given U(VI)-dendrimer molar ratio. The sealed centrifuge tubes were mixed on a rotary shaker for 120 min. Then, the solution was subsequently withdrawn from each equilibrated tube and transferred into a Millipore Centricon filter with a molecular weight cut-off of 5000 Da. The filters were centrifuged for 20 min at 3000 rpm to separate de U(VI)-laden dendrimers from the aqueous solutions. The concentration of U(VI) in each filtrate (*U*_0_) was measured by fluorescence spectrophotometry, for that, each sample was diluted 2-fold with a 10% wt of a H_3_PO_4_ solution. The complexation of U(VI) ions with phosphoric acid causes a large enhancement of their fluorescence emission intensity. This provides the basis of a U(VI) assay method with a detection limit of 40.0 ppb [30,38].

All fluorescence emission spectra were collected on a steady-state RF-5301pc Spectrofluorophotometer (Shimadzu, Kyoto, Japan) using an excitation wavelength of 280 nm. The emission spectra were recorded between 480 and 545 nm. The intensity of the emission peak at 508 nm was used to develop the U(VI) calibration curve. The concentration of U(VI) bound to a dendrimer (Ub) (mol/L) was expressed as follows:

(1) $$U_{b}=U_{c}-U_{0}$$

where *U_c_* is the U(VI) concentration in each centrifuge tube and *U*_0_ is the concentration of the U(VI) in the filtrate. The extent of binding (ExtB) (mol of U(VI) bound per mol of dendrimer), the concentration of dendrimer (*C_den_*) in solution (mol/L) and the fractional binding (*F_Bin_*) were expressed as follows:

(2) ExtB=UbCden

(3) Cden=mdVs.Mwd

(4) FBin=100 × (UbUc)

where *m_d_* (g) is the mass of the dendrimer in solution, *V_s_* (L) is the solution volume and *M_wd_* (g/mol) is the molar mass of the dendrimer [30].

### 3.4. Red Blood Cells (RBCs) interactions

#### 3.4.1. Sample Collection

Blood from healthy donors was extracted and anticoagulated with heparine tubes (BD Vacutainer, Becton Dickinson, Franklin Lakes, NJ, USA). Erythrocytes were separated from the plasma and leucocytes by centrifugation (1500 rpm, 5 min) at 4 °C and washed three times with phosphate-buffered saline (PBS: 150 mM NaCl, 1.90 mM NaH_2_PO_4_, 8.10 mM Na_2_HPO_4_, pH 7.4; Sigma Aldrich Co.). All the protocols were authorized by the ethic committee of the University of Talca in accordance with the Declaration of Helsinki (approved by the 18th World Medical Assembly in Helsinki, Finland, in 1964).

#### 3.4.2. Assay of Red Blood Cell Lysis

The hemolysis assay was performed according to the method of Duncan *et al.* [39]. Briefly, washed RBCs at 2% were incubated at room temperature with a concentration of 4 µM of the selected dendrimer. After 2 h of incubation, samples were centrifuged at 2000 rpm for 10 min and the absorbance of the supernatant was measured at 550 nm (Clima Plus, RAL S.A, Barcelona, Spain). Hemolysis was expressed as percentage of released hemoglobin, using as control (100% of hemoglobin released) a solution of RBCs incubated with Triton X-100 (0.2% *v*/*v*, Sigma Aldrich Co.). Additionally, morphological changes in the RBCs were determined by optical microscopy.

### 3.5. In Vivo Acute Toxicity Assay

#### 3.5.1. Animals

For the *in vivo* study a total of 48 male mice (C57BL/6) weighing 25–30 g purchased from the Chilean Public Health Institute were used. Animal housing and experiment protocols were approved by the University of Talca in accordance to Governmental Experimental Animal Administrative Regulations (CONICYT). All groups were maintained at a constant temperature (22 ± 1 °C) on a 12 h light/12 h dark cycle (lights-on at 08:00 am) and had free access to food and water.

#### 3.5.2. Animal Experimental Design

A single dose of 5.0 mg/kg of U(VI) as uranyl acetate (UO_2_(CH_3_COO)_2_·2H_2_O) in saline (0.9% NaCl), or the vehicle alone, was given intraperitoneal (ip). Eight groups of study were designed: the first group (*n =* 6) was designated as the control group (saline, ip); the second group (*n =* 6) for U(VI) acute intoxication (5.0 mg/kg of U(VI), ip); the third group (*n =* 6) for PAMAM G4-Lys-Fmoc-Cbz (16.0 mg/kg, ip); the fourth group (*n =* 6) for PAMAM G5-Cou (40.0 mg/kg, ip); the fifth group (*n =* 6) for ethylenediaminetetraacetic acid (EDTA, 75.0 mg/kg, ip); the sixth group (*n =* 6) for PAMAM G4-Lys-Fmoc-Cbz (16.0 mg/kg, ip) immediately after U(VI) administration (5.0 mg/kg, ip); the seventh group (*n =* 6) for G5-Cou (40.0 mg/kg) immediately after U(VI) administration (5.0 mg/kg, ip); the eighth group (*n =* 6) for EDTA (75.0 mg/kg, ip) immediately after U(VI) administration (5.0 mg/kg, ip). Animals were euthanized 48 h later and blood samples were collected.

### 3.6. Biochemistry and Nephrotoxicity

At the end of the experimental period, the mice were euthanized with ketamine-xylazine and blood samples were collected from the heart and the kidneys were removed. Plasma levels of urea, creatinine, LDH, uric acid and calcium were determined using commercially available kits according to the protocols provided by Valtek (Valtek Diagnostic, Nuñoa, Chile). Left kidneys were fixed in 10% neutral buffered formaldehyde, embedded in paraffin wax and automatically processed. Sections (3 μm in thickness) of the embedded tissue were stained with hematoxylin-eosin for observation under optic microscope.

### 3.7. Statistical Analysis

The *in vitro* data were obtained from three independent experiments and were analyzed with t test. Experimental groups were compared using a one-way analysis of variance (ANOVA) followed by Tukey test. Statistical tests were performed using GraphPad Prism 5 software, version 5.0 for Mac OS X; the results were expressed as mean ± SEM and *p*-values < 0.05 were considered significant.

## 4. Conclusions

This study shows that from the series of synthesized dendrimers only G4-Lys-Fmoc-Cbz and G5-Cou, at a concentration of 0.5 μM have a high capacity of U(VI) trapping without causing hemolysis or RBCs agglutination *in vitro*. Furthermore, it was demonstrated that the PAMAM G4-Lys-Fmoc-Cbz has a great ability to chelate the U(VI) *in vivo*, improving biochemical and histopathologic features caused by acute intoxication with uranium.
